# Klinische Studie PEPCA: Der Effekt standardisierter präoperativer Patientenedukation zu patientenkontrollierter Regionalanästhesie auf postoperative Schmerzen

**DOI:** 10.1007/s00482-023-00698-6

**Published:** 2023-02-07

**Authors:** Tobias Bacher, Andre Ewers

**Affiliations:** 1 Uniklinik für Anästhesiologie, perioperative Medizin und allg. Intensivmedizin, LKH Salzburg, Universitätsklinikum der Paracelsus Medizinischen Privatuniversität Salzburg (PMU), Müllner Hauptstr. 48, 5020 Salzburg, Österreich; 2 Koordination Klinische Pflegewissenschaft und -forschung, Uniklinikum Salzburg, Müllner Hauptstr. 48, 5020 Salzburg, Österreich; 3 Institut für Pflegewissenschaft und -praxis, Paracelsus Medizinische Privatuniversität Salzburg, Salzburg, Österreich

**Keywords:** Patientenschulung, Perioperative Versorgung, PCA, Akutschmerz, Pflege, Patient training, Perioperative care, Nursing, PCA, Acute pain

## Abstract

**Hintergrund:**

Die patientenkontrollierte Analgesie (kurz: PCA) stellt ein etabliertes Mittel zur postoperativen Schmerztherapie dar. Eine der möglichen Applikationsformen ist dabei die PCRA (patientenkontrollierte Regionalanästhesie), die Verabreichung eines Lokalanästhetikums mittels Regionalkatheter. Voraussetzung ist dabei, dass die Patienten eine entsprechende Einweisung in deren Einsatz erhalten. Zahlreiche Quellen empfehlen, diese vor der Op. durchzuführen, da präoperative Schulungsmaßnahmen zum Schmerzmanagement die postoperativen Schmerzen und das Wohlbefinden signifikant verbessern können.

**Fragestellung:**

Ziel dieser Studie war die Untersuchung des Effekts leitliniengestützter präoperativer Schulungen zu PCRA auf postoperative Schmerzen bei orthopädischen Eingriffen verglichen mit unstrukturierten postoperativen Einweisungen.

**Material und Methoden:**

Es wurde eine kontrollierte Interventionsstudie mit zwei randomisierten Gruppen durchgeführt. Insgesamt wurden 73 Patienten mit PCRA-Kathetern bei orthopädischen Eingriffen eingeschlossen. Die 37 Teilnehmer der Interventionsgruppe (IG) bekamen unmittelbar vor ihrem Eingriff eine leitfadengestützte, strukturierte Einschulung zum PCRA-Gebrauch sowie ein entsprechendes Handout. Die 36 Probanden der Kontrollgruppe (KG) erhielten eine unstrukturierte postoperative Einweisung im Aufwachraum. Schmerzen wurden anhand der numerischen Rangskala (NRS) 2 (t1), 6 (t2) und 24 h (t3) nach dem Eingriff erhoben.

**Ergebnisse:**

Zwar wies die IG zu t1 und t3 geringere durchschnittliche Schmerzen auf, jedoch konnten keine statistisch signifikanten Unterschiede zwischen den beiden Gruppen nachgewiesen werden.

**Diskussion:**

Anhand der Outcomes sind weiterführende Erhebungen mit adaptierten Stichprobengrößen und Erhebungszeitpunkten zu empfehlen.

## Abstract (en)

**Background:**

Patient-controlled analgesia (PCA) is a well-established form of postoperative pain management. One form of administration is patient-controlled regional analgesia (PCRA), where local anesthetics are administered via peripheral regional catheters; however, a prerequisite is that the patients are instructed on its use. A multitude of sources recommend that these instructions are given before surgery as preoperative training on pain management procedures has been shown to significantly reduce patients’ postoperative pain and increase their well-being.

**Objective:**

The aim was to assess the effect of guideline-assisted preoperative patient education for PCRA on postoperative pain in patients undergoing orthopedic surgery compared to unstructured postoperative standard instructions.

**Material and methods:**

A controlled study with two randomized samples and a postinterventional survey was conducted. Overall, 73 patients with PCRA catheters for orthopedic surgery were enrolled in the study. The 37 participants in the interventional group (IG) received guideline-based structured instructions on PCRA use as well as a handout immediately before the surgical intervention. The 36 patients in the control group (CG) received postoperative instructions in the anesthesia recovery room. Pain was documented according to the numerical rating scale (NRS) at 2h (t1), 6h (t2), and 24h (t3) after surgery.

**Results:**

There were no statistically significant differences in the average pain scores between the two groups; however, there were lower mean pain scores in the IG at t1 and t3.

**Conclusion:**

A significant reduction of pain in the IG could not be shown. Further studies concerning this topic with larger samples and adapted points in time are recommended.

## Einleitung

Über 80 % aller Patienten sind in den ersten 24–48 h nach einer Op. von mittleren bis starken Schmerzen betroffen [6, 7]. Eine unzureichende Schmerztherapie kann zu schwerwiegenden Komplikationen von Herz- und Lungenfunktion, dauerhaften Mobilitätseinschränkungen, Chronifizierung oder psychosozialen Beeinträchtigungen führen [6, 18]. Die Patienten selbst spielen dabei eine integrale Rolle in der Umsetzung eines suffizienten Schmerzmanagements. Sie sollten daher von Anfang an in die Strategien zur Schmerzbehandlung miteinbezogen werden [1, 4].

## Hintergrund und Fragestellung

### Allgemeines

Die patientenkontrollierte Analgesie (PCA) mittels Schmerzpumpe stellt ein seit vielen Jahren bewährtes Mittel zur sicheren Behandlung postoperativer Schmerzen dar. Ihre Applikations- und Einstellungsarten können je nach Notwendigkeit und Durchführbarkeit variiert werden. Die Applikation selbst erfolgt entweder systemisch/intravenös (PCIA) oder regional via Katheter (PCRA/PCEA; [19]).

Bei der Verwendung von PCA sollte vorab stets darauf geachtet werden, dass die Patienten deren Funktion verstehen und sie ohne Angst vor möglichen Nebenwirkungen korrekt anwenden können [12]. Die dabei zur Verfügung gestellten Informationen sollten sich mit Sinn und Zweck des PCA-Verfahrens, der richtigen Handhabung, Sicherheitsmerkmalen und Bedienungsmechanismen befassen [3].

In zahlreichen Publikationen, konnte der positive Effekt von präoperativen Patientenschulungen zum Schmerzmanagement auf die postoperative Schmerzreduktion gezeigt werden [2, 14, 15, 17, 20]. Einige Studien befassten sich speziell mit den Auswirkungen präoperativer PCA-Einweisungen [7, 9, 10, 16]. Wiederholt wird in diesen Studien dabei explizit auf die Wichtigkeit des Schulungszeitpunkts zugunsten der präoperativen Phase verwiesen. Das Augenmerk aller dieser Erhebungen lag dabei jedoch ausschließlich auf dem Umgang mit intravenöser PCIA [3, 5, 7, 9].

**Abb. 1:**
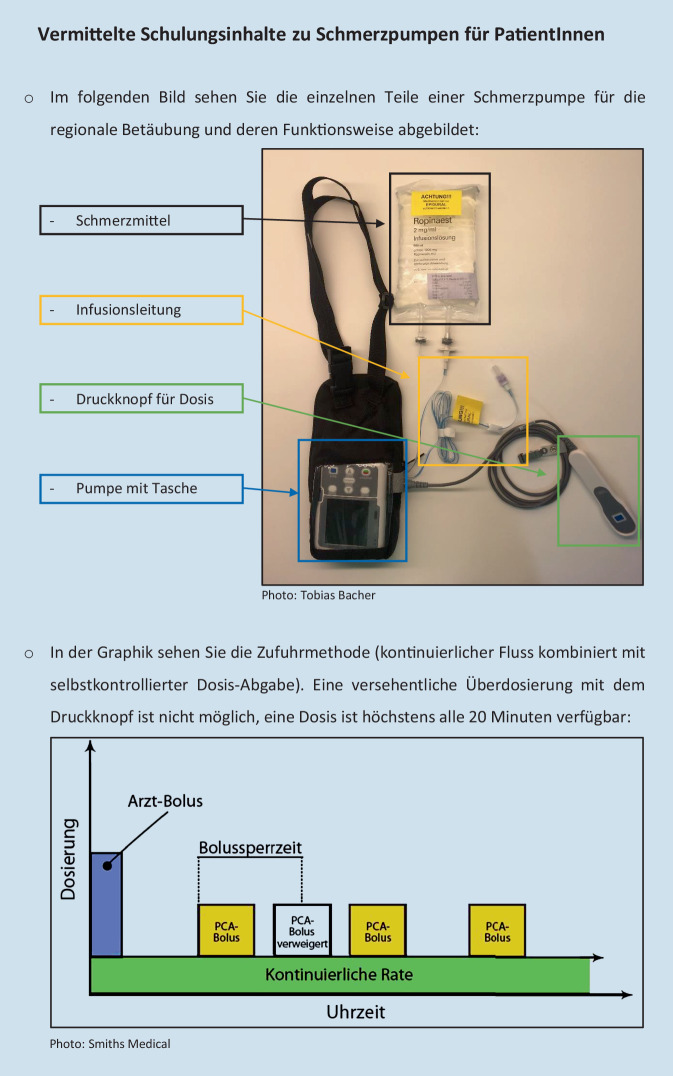
Handout für IG, S. 1

Im Vergleich zur PCIA unterscheidet sich die PCRA grundsätzlich in Hinsicht auf Pharmakokinetik und -dynamik der verwendeten Substanzen (meist Lokalanästhetika). Dementsprechend gilt es bei der PCRA-Anwendung auch auf ein konträres Spektrum an unerwünschten Nebenwirkungen zu achten. Auch ist die Katheteranlage eine vielfach komplexere Intervention – ultraschallgestützt und in unmittelbarer Nähe zu Nerven(-bündeln) –, wie auch die Katheterpflege. Weiters unterscheidet sich die PCRA in der Form des Verabreichungsregimes: Während bei PCIA ausschließlich auf initiative Betätigung der Pumpe ein Medikament abgegeben wird [19], liegt der PCRA ein kontinuierlicher Fluss des Lokalanästhetikums zugrunde, welcher ebenfalls bei Bedarf durch initiative Betätigung des Bolusgebers unterstützt werden kann (Abb. 1, grün markiert; [8]). Aus diesen Gründen scheint eine spezifische Einweisung in diese Applikationsform unerlässlich.

### Forschungsfrage

Die Fragestellung dieser klinischen Studie PEPCA (**P**atienten**e**dukation zu **PCA**) war, ob eine standardisierte und leitfadengestützte präoperative Patientenschulung für Schmerzpumpen zu signifikant geringeren postoperativen Schmerzen bei PatientInnen mit PCRA-/Plexuskathetern führt gegenüber unstrukturierten postoperativen Einweisungen.

## Studiendesign und Untersuchungsmethoden

### Design

Der vorliegenden klinischen Studie liegt ein randomisiertes, kontrolliertes Interventionsdesign mit zwei Teilnehmergruppen zugrunde.

### Stichprobe

Alle eingeschlossenen Patienten befanden sich zu elektiven Eingriffen an der Abteilung für Orthopädie und Traumatologie des Uniklinikums Salzburg. Die Rekrutierung fand am Tag des Eingriffs im OP-Vorbereitungsraum (*PACU*) statt. Nach schriftlichem Einverständnis wurden die Teilnehmer einer der beiden Studiengruppen randomisiert zugeteilt. Die Zuteilung erfolgte anhand einer *Microsoft-Excel*-Datei, in der 40 Nullen und Einsen per Zufall aneinandergereiht waren. Eins stand für Interventions- (IG), Null für Kontrollgruppe (KG). Die Zuteilung erfolgte durch die diensthabende Anästhesiepflege. Alle Teilnehmer erfüllten folgende Kriterien:

- 18 Jahre oder älter
- Ausreichende Deutschkenntnisse
- Verständnis von Ziel und Zweck der Erhebung
- Körperliche und geistige Fähigkeit zur Bedienung einer Schmerzpumpe
- Einverständnis zu einem PCRA-Verfahren

Einziges Ausschlusskriterium waren bereits bestehende bzw. chronische Schmerzen.

Die Stichprobengröße wurde mittels *G‑Power *ermittelt. Die Power (1−β) wurde mit 0,80 und α als 0,05 definiert. Die Effektgröße errechnete sich aus den Mittelwerten und Standardabweichungen der NRS-Werte einer ähnlichen Studie mit PCIA-Verfahren von Hong & Lee zu denselben Erhebungszeitpunkten [7]. Dies ergab eine Mindestgröße von 33 Teilnehmern pro Gruppe.

### Schulungsleitfaden und -materialien

Im Vorfeld zur Erhebung wurde ein standardisierter Leitfaden zur Patientenedukation entwickelt, welcher auf dem Konzept für *Mikroschulungen* der Universität Witten-Herdecke beruhte [13]. Die Inhalte des Schulungsleitfadens beruhten zum einen auf den Informationen von Patienten, welche vormalig eine PCRA erhalten hatten. Zum anderen wurde auf Informationsmaterial des PCA-Pumpen-Herstellers *Smiths Medical *zurückgegriffen [8]. Weiters kamen Inhalte einer Studie aus dem Jahr 1999 zum Einsatz, wobei die darin beschriebenen Informationen zu PCIA-Verfahren auf PCRA adaptiert wurden [9]. Die Geräte, welche zum Einsatz kamen, waren vom Typ *CADD-Solis *der Fa. *Smiths Medical*. Eine Pumpe in PCRA-Konfiguration diente als Anschauungsstück für die präoperativen Mikroschulungen.

### Instrument

Die Dokumentation der Daten erfolgte auf einheitlichen Bögen mit 10 Items. Die Items 1–8 umfassten Daten zu Alter, Geschlecht, Eingriffsart, -datum und -dauer sowie zur Lokalisation des PCRA-Katheters. Da die Schulungen der IG unmittelbar vor dem Eingriff und der PCRA-Legung stattfanden, musste die Möglichkeit einer retrograden Amnesie bezüglich der präoperativ vermittelten Inhalte beachtet werden, weshalb Item 9 zur Abfrage einer solchen im Aufwachraum integriert wurde. Das letzte Item erfasste die postoperativen Schmerzen anhand der numerischen Rangskala (NRS) 2, 6 und 24 h nach Op.-Ende. Diese Zeitpunkte wurden analog zur Studie von Hong & Lee, welche auch schon als Anhaltspunkt für die Fallzahlberechnung diente, gewählt [7].

### Durchführung der Datenerhebung

Die Erhebung fand vom Nov. 2018 bis Febr. 2019 statt. Insgesamt wurden 73 Patienten in die Studie eingeschlossen. 37 wurden der IG, 36 der KG zugeordnet.

Die Teilnehmer (kurz: TN) der KG erhielten die für den Standort übliche Behandlung. So wurde der PCRA-Katheter präoperativ im PACU gelegt, bevor die Patienten in den OP fuhren. Sie bekamen vorab keine weitere Einweisung in die Handhabung der PCRA. Nach OP-Ende wurden die TN an den Aufwachraum übergeben, wo durch das dortige Pflegepersonal eine mündliche Einweisung in die Verwendung der PCRA erfolgte, bevor sie auf die Bettenstationen zurücktransferiert wurden. Diese Einweisung erfolgte zum damaligen Zeitpunkt keiner konkreten Richtlinie folgend.

In der IG erhielten die Patienten im PACU eine ca. 15-minütige leitfadengestützte Mikroschulung durch die Anästhesiepflege. Zudem bekamen sie ein zweiseitiges Handout, welches alle Inhalte noch einmal zusammenfasste (Abb. 1 und 2). Dieses verblieb für die Dauer der gesamten PCRA-Therapie zum Nachlesen am Patientenbett. Erst wenn nach Abschluss der Einweisung keine weiteren Fragen für die TN mehr bestanden, wurde mit der Legung des PCRA-Katheters fortgefahren und die Patienten anschließend in den OP gebracht. Die Patienten wurden im Aufwachraum nicht mehr eingewiesen, aber vor Verlegung auf die Station gefragt, ob sie die präoperativ geschulten Inhalte rekapitulieren konnten. Falls nicht, erhielten sie eine erneute Einweisung und wurden aus der Studie ausgeschlossen.

**Abb. 2:**
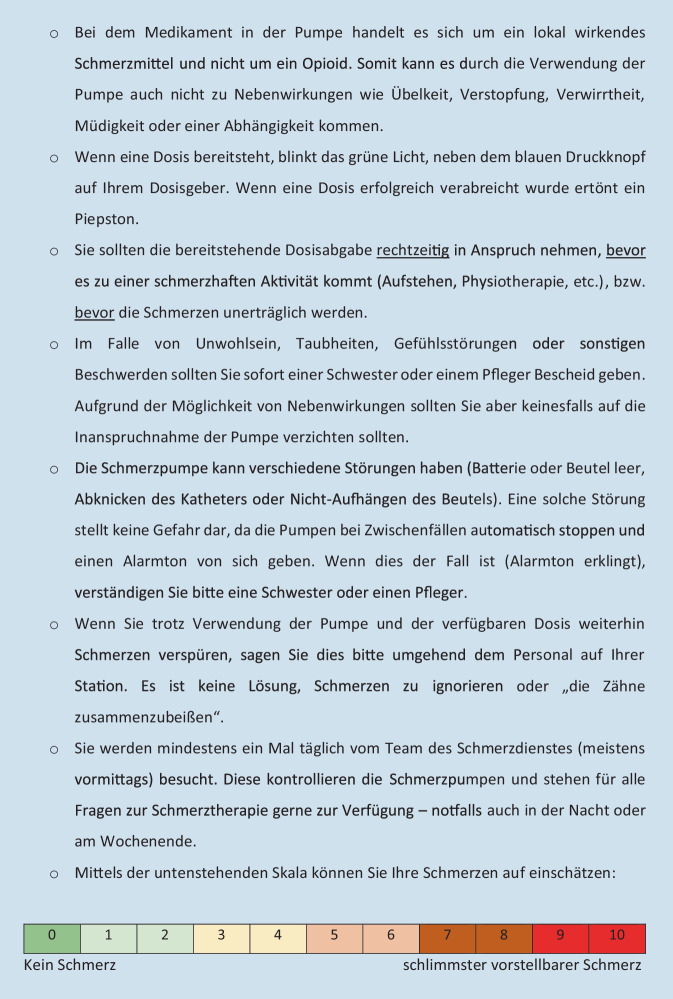
Handout für IG, S. 2

### Datenanalyse

Statistische Berechnungen erfolgten mittels *IBM SPSS Statistics, V.24*. Die Unterschiede zwischen den Mittelwerten wurden mittels Mann-Whitney-U-, Chi^2^- und t‑Test für unabhängige Stichproben analysiert und sind in Tab. 1, 2, 3 und 4 jeweils angeführt.

**Tab. 1:** Alter, Geschlecht und OP-Dauer

	IG (*n* = 37)	KG (*n* = 36)	Gesamt (*n* = 73)		
Variable	MW (SA) oder *n *(%)	MW (SA) oder *n *(%)	MW (SA) oder *n *(%)	t oder Chi^2^	*p*
*Alter*	62,97 (14,09)	62,89 (17,22)	62,93 (15,61)	−0,023	0,982
*Geschlecht*					
Weiblich	17 (45,9 %)	20 (54,1 %)	37 (50,7 %)	0,674	0,412
Männlich	20 (55,6 %)	16 (44,4 %)	36 (49,3 %)	–	–
*OP-Zeit in Minuten*	176,86 (66,11)	158,50 (55,08)	167,81 (61,20)	−1,29	0,202

*MW* Mittelwert, *SA* Standardabweichung, *p* Signifikanz

**Tab. 2:** Eingriffsart

	IG (*n* = 37)	KG (*n* = 36)	Gesamt (*n* = 73)		
*OP-Art*	*n (%)*	*n (%)*	*n (%)*	*Chi*^*2*^	*p*
ASK	10 (27,03 %)	10 (27,78 %)	20 (27,4 %)	1,008	0,799
Gelenksersatz	21 (56,76 %)	17 (47,22 %)	38 (52,05 %)	–	–
Osteosynthese	2 (5,41 %)	3 (8,33 %)	5 (6,84 %)	–	–
Sonstiges	4 (10,8 %)	6 (16,67 %)	10 (13,71 %)	–	–
*OP-Gebiet*					
Knie	13 (35,14 %)	16 (44,44 %)	29 (39,72 %)	1,797	0,407
Schulter	21 (56,76 %)	15 (41,67 %)	36 (49,32 %)	–	–
Sonstiges	3 (8,1 %)	5 (13,89 %)	8 (10,69 %)	–	–

*MW* Mittelwert, *SA* Standardabweichung, *p* Signifikanz

**Tab. 3:** PCRA-Verfahren

	IG (*n* = 37)	KG (*n* = 36)	Gesamt (*n* = 73)		
Katheterverfahren	*n* (%)	*n* (%)	*n* (%)	Chi^2^	*p*
N. femoralis	13 (35,14 %)	16 (44,44 %)	29 (39,72 %)	2,051	0,726
Interskalenär	21 (56,76 %)	17 (47,22 %)	38 (52,05 %)	–	–
N. ischiadicus	1 (2,7 %)	2 (5,56 %)	3 (4,12 %)	–	–
Infraklavikulär	1 (2,7 %)	1 (2,78 %)	2 (2,74 %)	–	–
Axillär	1 (2,7 %)	0 (0,00 %)	1 (1,37 %)	–	–

*MW* Mittelwert, *SA* Standardabweichung, *p* Signifikanz

**Tab. 4:** Postoperative Schmerzen lt. NRS

	IG	KG		t‑Test		Mann-Whitney-U-Test	
Zeitpunkt	MW (SA)	MW (SA)	MW-Diff.	T‑Wert	*p*	Mann-Whitney‑U	*p*
t1	0,57 (1,303)	0,78 (1,267)	0,21	0,699	0,487	578,000	0,224
t2	1,11 (2,135)	0,62 (1,256)	−0,49	−1,170	0,246	629,000	0,149
t3	2,07 (2,050)	2,72 (2,747)	0,65	1,009	0,317	553,000	0,068

*MW* Mittelwert, *SA* Standardabweichung, *p* Signifikanz

### Ethische Gesichtspunkte

Die Erlaubnis zur Durchführung wurde vorab von der zuständigen Ethikkommission des Bundeslands Salzburg erteilt. Zudem wurde die schriftliche Einverständniserklärung aller TN vor jeglicher studienbezogenen Intervention eingeholt. Eine Verblindung war nicht möglich, da die für das Einverständnis notwendige Studienbeschreibung Rückschlüsse auf die Gruppen zuließ. Jede Form der medikamentösen Prämedikation war bis zum Abschluss studienspezifischer Interventionen untersagt.

## Ergebnisse

### Demografie und Eingriffsdauer

Sämtliche Ergebnisse hierzu können Tab. 1 entnommen werden. Drop-outs waren weder während der Erhebungsphase noch nachträglich zu verzeichnen. Der jüngste Teilnehmer war 20 Jahre alt, der älteste 86. Das durchschnittliche Alter war in beiden Gruppen fast identisch (IG: 63,0; KG: 62,9). Die OP-Dauer wurde als Zeitraum zwischen Ein- und Ausschleusung definiert. Deren Spannweite reichte von 30 bis 315 min.

### Eingriffsarten

Mehr als die Hälfte der Eingriffe (52,1 %) waren Gelenkersätze, gefolgt von Arthroskopien (27,4 %). 6,8 % waren Osteosynthesen, die restlichen 13,7 % bestanden aus osteochondralen Abtragungen sowie offenen Sehnen- oder Bandnähten. Knapp 50 % betrafen die Schulter, gefolgt von Knien mit 39,7 %. Gut 10 % beliefen sich auf andere OP-Gebiete, wie Oberarm, Ellbogen, Schlüsselbein oder Fuß. Siehe dazu Tab. 2.

### Lokalisation der PCRA-Katheter

Das am häufigsten eingesetzte Verfahren stellte mit 52,1 % der interskalenäre Katheter dar. Zweithäufigstes (39,7 %) war der Femoraliskatheter und in 4,1 % wurde ein Plexuskatheter am N. ischiadicus gelegt. Nur 2‑mal (2,7 %) kam ein infraklavikulärer und ein einziges Mal ein axillärer Plexuskatheter zum Einsatz (Tab. 3).

### Retrograde Amnesie

Alle TN in der IG konnten die Schulungsinhalte postoperativ reproduzieren. Somit konnte eine retrograde Amnesie ausgeschlossen.

### Postoperativer Schmerz

Der Ruheschmerz anhand NRS in der IG belief sich auf 0,57 (t1), 1,11 (t2) und 2,07 (t3). Im Vergleich dazu zeigte die KG durchschnittliche Schmerzen von 0,78 (t1), 0,62 (t2) und 2,72 (t3). Die Unterschiede der Gruppen wurden mittels t‑ und Mann-Whitney-U-Test (aufgrund der geringen Stichprobengröße) untersucht. Dabei konnte kein signifikanter Unterschied in den Mittelwertdifferenzen zwischen KG und IG gezeigt werden. Alle Werte, Standardabweichungen und Signifikanzen sind in Tab. 4 dargestellt.

## Diskussion

**Abb. 3:**
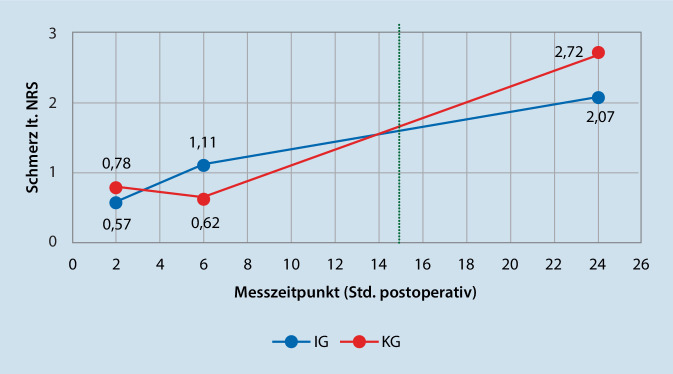
Postoperative Schmerzentwicklung über 24 h

Es konnte kein signifikanter Effekt von präoperativen Mikroschulungen zum Umgang mit PCRA auf die postoperativen Schmerzen gegenüber postoperativen Einweisungen gefunden werden. Abb. 3 zeigt die Mittelwerte anhand der NRS für beide Studiengruppen zu den drei Messzeitpunkten. Dabei ist ersichtlich, dass die Schmerzen in der IG in den ersten sechs Stunden zunehmen, die Kurve danach aber zusehend abflacht. Im Vergleich verläuft die Kurve der KG umgekehrt, zeigt also zuerst geringere Schmerzwerte, die aber bis t3 stetig ansteigen. Hervorzuheben ist dabei, dass beide Kurven sich nach 14–15 h kreuzen (grün strichlierte Linie), was entsprechend der Literatur [19] auch etwa jenem Zeitpunkt entspricht, an dem die Wirkung der Single-Shot-Analgesie, die im Zuge der Katheteranlage appliziert wird, nachlässt. Demnach könnte argumentiert werden, dass die Intervention der präoperativen Mikroschulungen in der IG ab jenem Zeitpunkt effektiv wurde, zu dem die initiale Analgesie nachließ und von der patienteneigenen PCRA-Bedienung bzw. Bolusgabe abhängig wurde.

Die Tatsache, dass kein einziger Fall in der IG von einer retrograden Amnesie berichtete, kann als Indiz gewertet werden, dass keine Kontraindikationen für unmittelbar präoperativ vorgenommene Schulungen besteht. Dies ist insbesondere vor dem Hintergrund wichtig, dass die Entscheidung zu einem adjuvanten PCRA-Verfahren oft sehr kurzfristig getroffen wird.

### Vergleich mit anderen Studien

Bezüglich der Stichprobengröße war PEPCA vergleichbar mit anderen hier bereits zitierten Studien [7, 9, 10, 16]. Ein Vergleich der Outcomes mit der Literatur schien zum Zeitpunkt der ersten Analyse der Daten schwierig, da sich keine der bis dahin erschienenen Publikationen mit PCRA-Verfahren beschäftigt hatte. Ein 2008 veröffentlichtes systematisches Review kam zu keinem eindeutigen Ergebnis. Dort konnte nur eine von sechs Studien einen positiven Effekt strukturierter präoperativer Schulungen zu PCIA nachweisen [21]. Erst 2019 erschien eine Studie, bei der die Intervention mittels Femoraliskatheter bei Knieersatz untersucht wurde. Aber auch hier ist die Vergleichbarkeit nur bedingt möglich, da der Effekt mittels VAS, anstatt NRS, und ausschließlich bei ein- und demselben Verfahren untersucht wurde. Hierbei allerdings mit signifikant geringeren Schmerzen in der IG [11].

### Limitationen

Verglichen mit anderen Studien hatte PEPCA eine heterogenere Stichprobe. So stellte, unabhängig von Alter, Geschlecht oder Vorerkrankungen, nur eine bereits vorab bestehende Schmerztherapie ein Ausschlusskriterium dar. Auch wurde nicht in Bezug auf die Art des Eingriffs differenziert, was sich u. a. in der großen Spannweite bei der OP-Dauer widerspiegelt. Ebenso war der Umfang der gesammelten Daten durch das sehr kleine Studienteam limitiert. So konnten zusätzliche Parameter, wie die Anzahl der angeforderten Boli, Nebenwirkungen, Rescue-Medikationen etc., nicht erhoben werden [7, 9, 10]. Für zukünftige Erhebungen wären aus Sicht der Autoren daher folgende Punkte zu berücksichtigen:

- Homogenere Stichproben oder Vergrößerung der Fallzahl
- Differenzierung hinsichtlich Invasivität und Dauer der chirurgischen Eingriffe
- Miteinbeziehung weiterer Größen wie Anzahl der abgegebenen Boli, zusätzlich verabreichter Analgetika etc.
- Anpassung der Messzeitpunkte, sodass die erste Messung erst bei Nachlassen des „single shot“ erfolgt, dafür aber mit verlängerter Beobachtungszeit über die ersten 48 h postoperativ, analog zu Lin et al. [11]

Umfang und Inhalt der Einweisungen in der KG sind – aufgrund der damals fehlenden Standardisierung im regulären Krankenhausbetrieb – retrospektiv nicht nachvollziehbar. Daher ist eine inhaltliche Ähnlichkeit zu den leitfadengestützten Schulungen nicht gänzlich auszuschließen.

## Fazit für die Praxis

- Eine Handlungsempfehlung kann weder für noch gegen eine präoperative Patientenschulung in der PCRA-Bedienung bei orthopädischen Eingriffen ausgesprochen werden.
- Eine Flexibilisierung des Schulungszeitpunkts zur Einweisung in PCA-Verfahren ist unabhängig von prä- oder postoperativer Phase möglich.
- Es besteht ein definitiver Bedarf für weitere Erhebungen zu der hier behandelten Fragestellung, unter Beachtung obiger Limitationen.

## References

[1] Blumenberg P, Krebs M, Moers M, Stehling H, Stomberg D (2020) Expertenstandard Schmerzmanagement in der Pflege. Schriftenreihe des Deutschen Netzwerks für Qualitätsentwicklung in der Pflege. Hochschule Osnabrück Fakultät für Wirtschafts- und Sozialwissenschaften, Osnabrück

[2] Chen S‑R, Chen C‑S, Lin P‑C (2014) The effect of educational intervention on the pain and rehabilitation performance of patients who undergo a total knee replacement. J Clin Nurs 23(1–2):279–287. 10.1111/jocn.12466 24313941 10.1111/jocn.12466

[3] Clifford T (2013) Patient controlled analgesia—safe practices. J Perianesth Nurs 28(2):113–114. 10.1016/j.jopan.2013.01.001 23522273 10.1016/j.jopan.2013.01.001

[4] Cox F (2010) Basic principles of pain management: assessment and intervention. Nurs Stand 25(1):36–39. 10.7748/ns2010.09.25.1.36.c7983 20949749 10.7748/ns2010.09.25.1.36.c7983

[5] Deutsche Interdisziplinäre Vereinigung für Schmerztherapie (DIVS) e. V. (2009) S3-Leitlinie „Behandlung akuter perioperativer und posttraumatischer Schmerzen“ (S3-Guideline for the ttreatment of acute peri-operative and post-traumatic pain)

[6] Ewers A (2016) Who cares? Warum die Patienteneinbindung im postoperativen Schmerzmanagement so wichtig ist. Lecture at the Austrian Nursing Congress 2016, Vienna

[7] Hong S‑J, Lee E (2012) Effects of a structured educational programme on patient-controlled analgesia (PCA) for gynaecological patients in South Korea. J Clin Nurs 21(23–24):3546–3555. 10.1111/j.1365-2702.2011.04032.x 22624870 10.1111/j.1365-2702.2011.04032.x

[8] Ketchum D, Krause JV (2014) CADD® Solis 2100, 2110 Tragbare Infusionspumpe. Bedienerhandbuch Version 3.0

[9] Knoerl DV, Faut-Callahan M, Paice J, Shott S (1999) Preoperative PCA teaching program to manage postoperative pain. Medsurg Nurs 8(1):25–33, 36 10232210

[10] Lam KK, Chan MTV, Chen PP, Kee WDN (2001) Structured preoperative patient education for patient-controlled analgesia. J Clin Anesth 13(6):465–469. 10.1016/S0952-8180(01)00304-X 11578894 10.1016/s0952-8180(01)00304-x

[11] Lin X, Zhou Y, Zheng H, Zhang J, Wang X, Liu K, Wang J, Guo X, Li Z, Han B (2019) Enhanced preoperative education about continuous femoral nerve block with patient-controlled analgesia improves the analgesic effect for patients undergoing total knee arthroplasty and reduces the workload for ward nurses. BMC Anesthesiol 19(1):150. 10.1186/s12871-019-0826-3 31409300 10.1186/s12871-019-0826-3 PMC6693176

[12] Nardi-Hiebl S, Eberhart LHJ, Gehling M, Koch T, Schlesinger T, Kranke P (2020) Quo vadis PCA? A review on current concepts, economic considerations, patient-related aspects, and future development with respect to patient-controlled analgesia. Anesthesiol Res Pract 2020:9201967. 10.1155/2020/9201967 32099543 10.1155/2020/9201967 PMC7040376

[13] Netzwerk Patienten- und Familienedukation e. V. (2017) Mikroschulungen. Universität Witten/Herdecke. https://patientenedukation.de/materialien/mikroschulungen. Zugegriffen: 1. Juli 2018

[14] Porras-González MH, Barón-López FJ, García-Luque MJ, Morales-Gil IM (2015) Effectiveness of the nursing methodology in pain management after major ambulatory surgery. Pain Manag Nurs 16(4):520–525. 10.1016/j.pmn.2014.09.013 25530124 10.1016/j.pmn.2014.09.013

[15] Sayin Y, Aksoy G (2012) The effect of analgesic education on pain in patients undergoing breast surgery: within 24 hours after the operation. J Clin Nurs 21(–10):1244–1253. 10.1111/j.1365-2702.2011.04009.x 22404338 10.1111/j.1365-2702.2011.04009.x

[16] Shovel L, Max B, Correll DJ (2016) Increasing patient knowledge on the proper usage of a PCA machine with the use of a post-operative instructional card. Hosp Pract 44(2):71–75. 10.1080/21548331.2016.1149015 10.1080/21548331.2016.1149015 26837536

[17] Smith MY, DuHamel KN, Egert J, Winkel G (2010) Impact of a brief intervention on patient communication and barriers to pain management: results from a randomized controlled trial. Patient Educ Couns 81(1):79–86. 10.1016/j.pec.2009.11.021 20036097 10.1016/j.pec.2009.11.021

[18] Thoma R (2014) Epidemiologie akuter und chronischer Schmerzes. Fortbildung Bayerischer Schmerzeinrichtungen, Curriculum Spezielle Schmerztherapie, Großhadern, Deutschland. https://silo.tips/download/epidemiologie-akuter-und-chronischer-schmerzes. Zugegriffen: 22. Sept. 2020

[19] Viscusi ER (2008) Patient-controlled drug delivery for acute postoperative pain management: a review of current and emerging technologies. Reg Anesth Pain Med 33(2):146–158. 10.1016/j.rapm.2007.11.005 18299096 10.1016/j.rapm.2007.11.005

[20] Wong EM‑L, Chan SW‑C, Chair S‑Y (2010) Effectiveness of an educational intervention on levels of pain, anxiety and self-efficacy for patients with musculoskeletal trauma. J Adv Nurs 66(5):1120–1131. 10.1111/j.1365-2648.2010.05273.x 20337801 10.1111/j.1365-2648.2010.05273.x

[21] Yankova Z (2008) Patients’ knowledge of Patient Controlled Analgesia (PCA) and their experience of postoperative pain relief: a review of the impact of structured preoperative education. J Perioper Nurs 3(3):91–99

